# Lightweight Building of an Electroencephalogram-Based Emotion Detection System

**DOI:** 10.3390/brainsci10110781

**Published:** 2020-10-26

**Authors:** Abeer Al-Nafjan, Khulud Alharthi, Heba Kurdi

**Affiliations:** 1 Computer Science Department, Imam Muhammad ibn Saud Islamic University, Riyadh 11432, Saudi Arabia; annafjan@imamu.edu.sa; 2 Computer Science Department, King Saud University, Riyadh 11543, Saudi Arabia; m.khloud@tu.edu.sa; 3 Computer Science Department, Taif University, Taif 26571, Saudi Arabia; 4 Mechanical Engineering Department, Massachusetts Institute of Technology (MIT), Cambridge, MA 02139, USA

**Keywords:** brain–computer interface (BCI), electroencephalogram (EEG), EEG-based emotion detection, spiking neural network, NeuCube

## Abstract

Brain–computer interface (BCI) technology provides a direct interface between the brain and an external device. BCIs have facilitated the monitoring of conscious brain electrical activity via electroencephalogram (EEG) signals and the detection of human emotion. Recently, great progress has been made in the development of novel paradigms for EEG-based emotion detection. These studies have also attempted to apply BCI research findings in varied contexts. Interestingly, advances in BCI technologies have increased the interest of scientists because such technologies’ practical applications in human–machine relationships seem promising. This emphasizes the need for a building process for an EEG-based emotion detection system that is lightweight, in terms of a smaller EEG dataset size and no involvement of feature extraction methods. In this study, we investigated the feasibility of using a spiking neural network to build an emotion detection system from a smaller version of the DEAP dataset with no involvement of feature extraction methods while maintaining decent accuracy. The results showed that by using a NeuCube-based spiking neural network, we could detect the valence emotion level using only 60 EEG samples with 84.62% accuracy, which is a comparable accuracy to that of previous studies.

## 1. Introduction

Brain–computer interfaces (BCIs) are technologies used to provide a direct interface between sensors and the brain. BCIs have been identified as an emerging technology in many trend reports [1,2], and the number of BCI devices is expected to grow rapidly [3]. BCIs use responses from brain activities in humans or animals to activate external devices. The feedback delivered and captured by these devices bypasses the motor functions of a user or subject, thereby preventing interference. In this regard, nascent brain activities are objectively translated to this device, which in turn interprets them.

**Figure 1:**
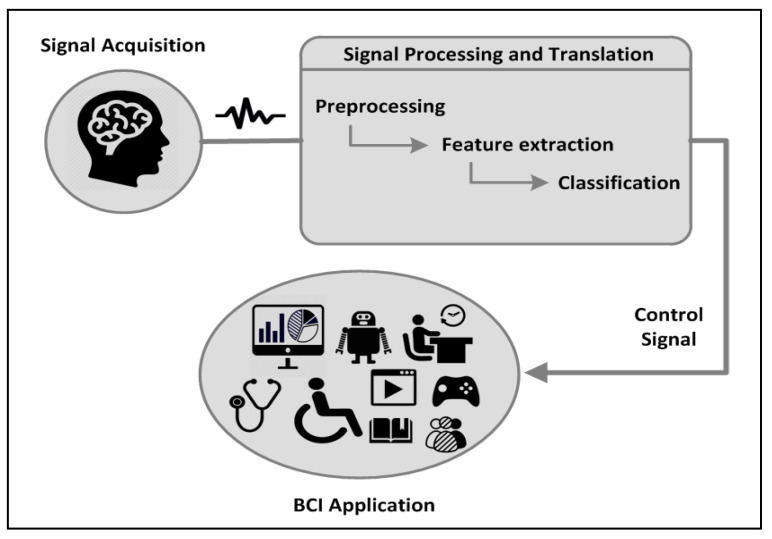
Basic components of a brain–computer interface.

Many BCI systems and platforms have been developed and deployed over the past decade. However, most platforms were implemented based on proprietary solutions or developed to solve specific domain problems. In an interdisciplinary field of research, BCI studies have sought to leverage recent advances in its interdisciplinary relationship with other fields and foster a range of applications in various fields, such as signal processing, neuroscience, machine learning, and information technology. Figure 1 illustrates the basic components of a BCI system.

BCI systems are designed to translate brain activities into control commands using a device that stimulates brain activity, thereafter providing an assessment of neurological function or sensory feedback. Sensory feedback is recorded, decoded, and eventually translated into a measurable neurophysiological signal, effector action, or behavior. Basically, any BCI system can be described by traditional processing pipelines, which include the following processing stages [1]:

1. Signal acquisition: This step deals with the measurement of brain signals using a particular type of sensor device. Furthermore, it involves different noninvasive neuroimaging methods that have been employed in BCI studies. These noninvasive techniques include electroencephalography (EEG), magnetoencephalography (MEG), functional magnetic resonance imaging (fMRI), functional near infrared (fNIR), and positron emission tomography (PET). All these methods have been reported as accepted. and safe imaging procedures used in research facilities and hospitals in clinical and nonclinical contexts. However, EEG is used most often in BCI research experimentation because the process is noninvasive to the research subject and minimal risk is involved. Moreover, the devices’ usability, reliability, and cost-effectiveness, along with the relative convenience of conducting studies and recruiting participants afforded by portability, have been cited as factors influencing the increased adoption of this method in applied research contexts. Yet, these advantages are often accompanied by challenges such as low spatial resolution and difficulty in managing signal-to-noise ratios.
2. Signal processing and translation: This BCI process includes the following steps:
  – Signal preprocessing: This step deals with the filtering of acquired signals and removal of noise. Basically, signals are amplified, filtered, digitized, and transmitted to a computer.
  – Feature extraction/selection: This step deals with the analysis of digital signals to discriminate relevant signal characteristics. The signals are then represented in a compact form suitable for translation into output commands through selecting a subset and reducing the dimensionality of features.
  – Feature classification: The subsequent signal features are fed into the feature translation algorithm, which translates the features into a control signal for the output device or into commands that accomplish the user’s intent.
3. Application/ feedback: The control signal from the signal processing and translation stage causes changes in the environment, device, or feedback mechanism of the EEG system. Many EEG-based BCI systems have been developed that have targeted different applications and shared the same goal of translating users’ intent into actions without using peripheral nerve impulses and muscles.

Recently, EEG-based emotion detection has garnered increased attention in both research and applied settings. It cuts across several application areas, including medical and nonmedical, and several disciplines such as clinical neurology, rehabilitative therapy, neurobiology, electronic engineering, psychology, computer science, medical physics, and biomedical engineering [3]. Most of these studies have proposed building an EEG-based emotion detection system with an EEG dataset larger than 300 samples, which has involved several feature extraction methods; by contrast, in the present study, we aim to propose a building process for an EEG-based emotion detection system that is lightweight in terms of a smaller EEG dataset size and with no involvement of feature extraction methods—all without compromising accuracy.

EEG-based emotion detection has promising potential applicability in numerous fields, where the ability to build such applications requires a sufficient size of EEG dataset and different feature extraction methods. This emphasizes the demand for a lightweight building process for an EEG-based emotion detection system with a smaller EEG dataset size and no feature extraction methods involved. Thus, investigations on the feasibility of promising tools and algorithms to maintain the high accuracy of EEG-based emotion detection systems—while learning from a smaller-sized preprocessed EEG dataset with no feature extraction methods involved—are highly encouraged.

Spiking neural networks (SNN) is the third generation of neural networks. It more closely mimics natural neural networks than artificial neural networks (ANN), as a neuron in SNN will fire when the accumulated stimuli reach above a threshold value, which makes SNNs more biologically realistic than ANNs [4].

Inspired by the fast information processing of spiking neural networks (SNNs) [5] as well as the results of studies that have used a NeuCube-based SNN as a classifier, which is an SNN architecture for spatio- and spectro-temporal brain data proposed in [6,7], we proposed an EEG-based emotion detection system that uses a NeuCube-based SNN as a classifier, with an EEG dataset of fewer than 100 samples [5,7,8]. The method was applied to address the following objectives: (i) to investigate how the SNN architecture NeuCube’s connectivity, learning algorithms, and visualization of the learning processes inside the SNN helped in classification and analysis of functional changes in brain activity when using EEG technique to recognize emotion states; (ii) to build an EEG-based emotion detection system using an EEG dataset of fewer than 100 samples and no feature extraction methods, with a NeuCube-based SNN as a classifier, and without compromising accuracy. Overall, our proof-of-concept work towards emotion detection and classification using NeuCube-based SNN shows promise and will provide the basis for continued research in this direction.

The remainder of this paper is organized as follows: Section 2 introduces the main concepts of this study with background details; Section 3 explores and reviews related studies in the field of EEG-based emotion detection and SNN-based EEG classification; Section 4 illustrates our proposed system, the acquisition of EEG data, the experiments, and the evaluation design; Section 5 reports the experimental results; and Section 6 presents the conclusion.

## 2. Background

In this section, we introduce an overview of EEG-based emotion detection systems and applications, SNNs, and NeuCube-based SNNs.

### 2.1. EEG Correlates of Emotion

Emotions play a critical role in human interactions with the outer world and are considered an important factor in human actions and behaviors. Therefore, the detection and recognition of emotion-related information have become attractive research topics, such as in human–computer interaction (HCI) and affective computing studies and have opened a wide space for multiple applications. Such applications range from visualizing a user’s emotional state to initializing a computer action based on this emotion, e.g., verbal feedback, or stimulating mini-games to initialize an application-dependent action, e.g., an e-learning application that can adjust the course to the current emotional state of the student [3,9].

Emotions can be measured objectively using various approaches, such as speech, gesture, body posture, or facial feature analyses, and also through physiological measurements that take advantage of sensors in direct contact with the user’s body, such as heart rate, skin temperature, skin conductivity, and brain activity. One advantage of using physiological measurements to indicate the emotional state of users is that users lack the ability to manipulate their emotional state, which is hidden in these measurements, as they can do with other approaches [9].

**Figure 2:**
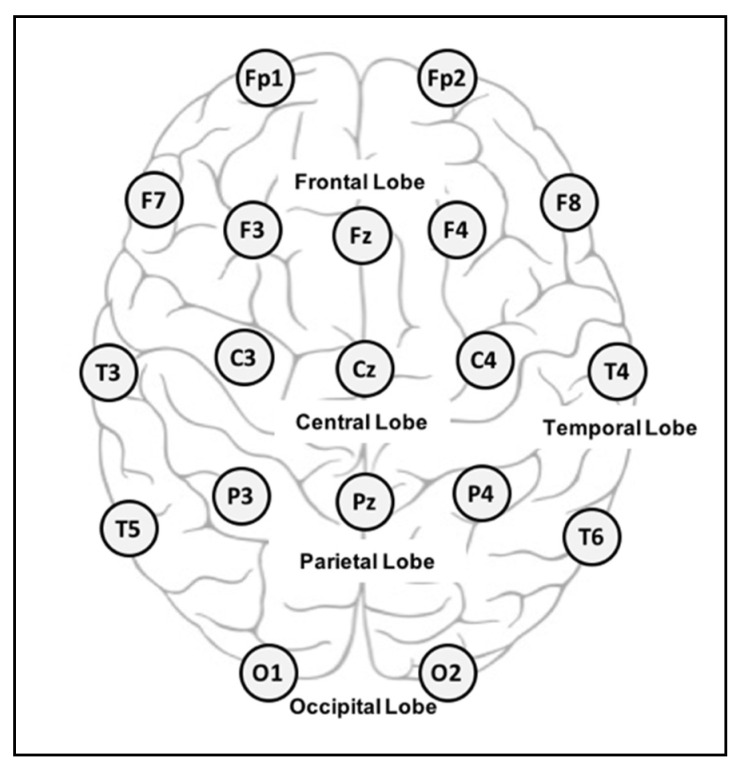
10–20 system of electrode placement.

**Figure 3:**
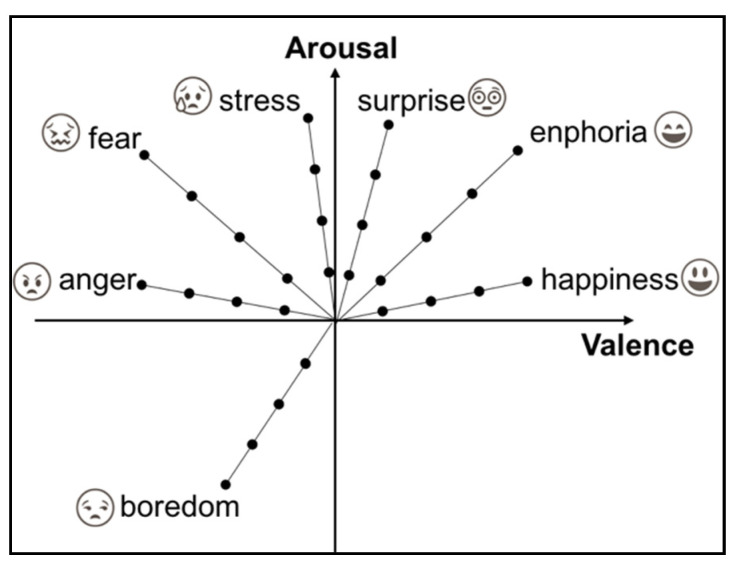
Arousal–valence emotional model.

EEG largely captures electrical activity by means of electrodes placed at specific locations on the scalp [10], following the international 10–20 system depicted in Figure 2. The emotional state that EEG-based emotion detection systems are intended to recognize is usually defined according to the 2-D arousal–valence emotional model; this model, proposed by Russell (1980), can map discrete emotion labels into the arousal–valence coordinate system [3], as shown in Figure 3.

### 2.2. EEG-Based Emotion Detection Applications

EEG-based emotion detection has been applied in numerous medical and non-medical fields. Consequently, numerous EEG-based BCI emotion detection systems and application have been developed that target different application areas. These applications are employed for both healthy individuals and unhealthy study participants. They all share the same research approach of using objective methods to determine affective emotional states.

In the following, we provide an overview and recent examples of applications that have been developed and studied for EEG-based emotion detection systems, categorizing them into five main areas including Medical and healthcare, Recreation, leisure and entertainment, Marketing, BCI aid and assistance, and Cognitive load estimation.

Medical and healthcare: This category includes research work that studies participants with medical conditions by comparing their affective state with the control group and experiments are conducted in clinical settings. These studies have suggested novel approaches for assisting, enhancing, mentoring, and diagnosing debilitative conditions of the study participants by using an EEG-based emotion detection technique. Medically related studies have detailed how EEG-based emotion detection systems are used to understand, diagnosis, and assess medical conditions. They have explored the relationship between symptoms and affective states in medical conditions such as disorder of consciousness [11], schizophrenia [12], Parkinson’s disease [13], and autism [14,15].

Recreation, leisure and entertainment: This category included studies that proposed EEG-based emotion detection in gaming and other associated entertainment domains. For example, they were used for observing the relationship between multimedia data (music/video) and human emotions. The results were then used to explore the effects of these multimedia data on emotional affective states for different gender and age groups. In gaming research, some work sought to detect gamers’ affective states in order to adapt to specific game’s features such as level of difficulty, punishment, and encouragement. All of these were investigated using an EEG-based emotion detection system. Research groups in the HCI community have started pushing boundaries by trying to determine the extent of their ability to peer into a user’s brain, if even roughly. Considering the minimally invasive nature of these devices and how they barely interfere with the day-to-day activities of a physically challenged person, researchers decided to further push the boundaries to ascertain the advantages that may accrue following what insight they may get in different application areas. Studies have explored BCI controlled recreational applications, such as games [16], virtual reality [17], brain-controlled art and music [18,19], and multimedia data tagging system [20,21].

Marketing: This category included studies that sought to understand consumer responses to market stimuli. During sales, a customer’s emotions can be strongly influenced by the perception of his/her surroundings. Recognition of emotional responses reveal true consumer preferences and can improve and assist the (buying) process. An EEG-based emotion detection system helps in the marketing of products to potential buyers by offering personalized information to the user through an understanding of individual preferences. These individualized preferences can then be extrapolated to other customers with a view to making tailored advertising preferences [22,23].

BCI aid and assistance: These research studies aimed to explore how assistive technologies become more effective by recognizing the affective states of persons. Assistive research studies were used to identify skills, user experience, and limitations of potential users. They were also used to improve behavior, cognition, and emotion regulation. BCI studies began as a move towards incorporating assistive technological solutions for persons with significant physical disabilities. It further drew research enthusiasts when it became apparent that persons with physical disabilities wanted to communicate with others, as well as have some level of control over their environment. The answer was to create computer-based recreational activities. Integrated emotion recognition systems with assistive technologies make them more efficient by recognizing affective state. BCIs were further used to identify users’ skills, experience, or limitations. Conversely, they were used to improve behavior, cognition, and emotion regulation [24,25].

Cognitive load estimation: This research work investigated how emotional states support learning processes. They assess an individual’s degree of engagement, attention, and cognitive load under different conditions. They also determined how emotions affect the way users consume information in school by assessing their levels of engagement and learning. These studies sought to monitor the alertness of individuals in job performance, security-critical tasks, measuring and estimating alert levels of cognitive load during task performance. Emotion is one of the key elements involved in learning and education; it also affects decision-making, communication, and the ability to learn. Research studies found that an emotional state has the potential to influence one’s thinking. Hence, measuring and estimating alertness levels and cognitive load during task performance can be applied in education and learning. This is done so as to assess the degree of engagement, attention, and cognitive load of a user under different conditions. Such cognitive tasks as listening to a lecture, solving logical problems, or workload assessments can benefit from BCI technologies [26,27].

### 2.3. NeuCube-Based Spiking Neural Networks

SNNs are considered the third generation of neural networks. The main difference between this generation and previous generations is the timing of potential spike firing of a neuron, which mainly depends on the spikes of other neurons. Depending on the input of the spiking neuron, it fires a spike or action potential at certain points in time, which could induce the postsynaptic potential of other spiking neurons according to an impulse response function; thus, information in an SNN is propagated by the timing of individual spikes. The input of the SNN must first be coded into sequences of neuronal spikes (spike trains) [28]. SNNs require a special learning rule, such as spike-timing-dependent plasticity (STDP), which adjusts the strength of a weight in response to the time difference between the pre- and postsynaptic spikes [29]. A significant number of researchers have used SNNs for different EEG-based pattern recognition problems and related them to the nature of information propagation in the SNN, which depends on the firing time of the spike and is close in nature to EEG data [30,31]. This makes SNNs an interesting method because they represent time and space compactly, process information rapidly, and can provide both time- and frequency-based information representation.

SNNs only accept data in forms of spike trains, thus input data should be first encoded into this form using special coding methods. Neurons in SNNs have the ability to generate action potentials or spikes when the pre-synaptic neurons exceed a threshold. Different neurons models have attempted to emulate this semi-biological behavior of SNN such as the spike response model (SRM), the Izhikevich neuron model, and the leaky integrated-and-fire (LIF) neuron. This behavior allows the weights of the SNN to increase if action potentials generated by the neurons decrease, otherwise based on the spike trains which induce the learning process of the SNN [4].

**Figure 4:**
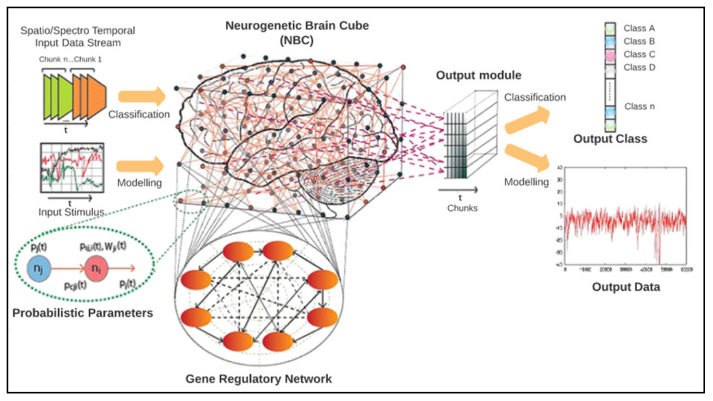
NeuCube model (Source: Kasabov 2012 [6]).

NeuCube is an SNN architecture for spatio- and spectro-temporal brain data, shown in Figure 4. The NeuCube architecture was first proposed by Nikola Kasabov for brain data modelling in [6] and was further developed as a multi-modular software/hardware system for large scale applications [5]. Basically, NeuCube-based SNN classification can be conducted in four steps:

1 Data encoding, in which EEG samples are converted into spike trains using encoding methods such as threshold-based representation (TBR), which generates a spike train whenever changes in the EEG data exceed a specific threshold.
2 SNN cube initialization, in which each neuron in the SNN cube is connected to its nearby neurons, which are within a specific distance threshold.
3 Training the cube using unsupervised learning, in which EEG data in the forms of spike trains are used to modify the initially set connection weights; thus, the same groups of spiking neurons in the SNN cube will be activated when similar input stimuli are presented.
4 Classification, in which the same dataset used for the unsupervised training phase is propagated to the SNN cube to train neurons in the output layer classifier. For each sample, a new neuron in the classifier is generated, and the state of the SNN cube is measured to establish a connection between this new neuron and a specific group of spiking neurons in the SNN cube. The state of the SNN cube is measured to establish a connection between this new neuron and a specific group of spiking NeuCube parameters, such as the SNN learning rule rate, the encoding method threshold, and the spike firing rate, which can affect the classification accuracy; therefore, an optimization module is proposed in this architecture to obtain the values of some of these parameters, which could lead to enhanced accuracy. The weight of this connection adapts according to the datasets used to link each sample, represented as a neuron in the output layer to specific emotional stimuli (emotion class), represented as a specific group of spiking neurons in the SNN cube.

## 3. Related Works

Several studies have investigated the contribution of various feature selection and extraction methods as well as classification algorithms to the overall performance of EEG-based emotion detection systems. Although the number of research studies on EEG-based emotion recognition has been increasing in recent years, EEG-based emotion recognition still faces challenges, including low signal-to-noise ratios (SNRs), nonstationary signals, and high inter-subject variability that limits effective analysis and processing [32].

In [33], the researchers conducted an experimental study in which they applied EEG-based emotion detection in clinical settings. A total of 26 subjects participated in a music-listening experiment where their EEG was recorded using 32 electrodes while they listened to 16 music clips (30 s each) as stimuli. A total of 416 samples were each labeled with one of the following four emotion classes: joy (positive valence and high arousal), anger (negative valence and high arousal), sadness (negative valence and low arousal), or pleasure (positive valence and low arousal), which followed the 2-D valence–arousal emotion model. The EEG samples were classified using two learning models, namely multilayer perceptron (MLP) and support vector machine (SVM), with four feature types: the power spectrum density of all 30 channels (PSD30), power spectrum density of 24 channels (PSD24), differential asymmetry of 12 electrode pairs (DASM12), and rational asymmetry of 12 electrode pairs (RASM12), computed on each one of the five frequency ranges of the EEG signals. The experimental results showed that SVM obtained the best averaged classification accuracy of 82.29%.

In [10], the researchers developed four learning models, and power spectral features and power spectral asymmetry were extracted from the DEAP dataset. These features fed into all four learning models, which had a deep learning network (DLN) as their main component. The proposed DLN consisted of three hidden layers, an input layer, and three neuron output layers where the three neurons corresponded to the three levels of arousal or valence that had been classified separately in the study. The first and second learning models consisted of a DLN with 100 and 50 hidden nodes in each layer, respectively, whereas the third learning model used principal component analysis (PCA) to reduce the number of features before feeding them into the DLN with 50 hidden nodes. The last model had the same structure as the third learning model except for its use of the covariate shift adaptation of principal components (CSA) to normalize each input feature, with the average of the previous feature values within a specific rectangular window, before applying PCA. The last model achieved the best classification accuracies for classifying the three levels of valence and arousal of 53.42% and 52.05%, respectively.

In [34], the researcher proposed a real-time emotion classification system. Four emotional classes—low arousal/low valence (LALV), low arousal/high valence (LAHV), high arousal/low valence (HALV), and high arousal/high valence (HAHV)—were classified with accuracies of 84.05% for arousal levels and 86.75% for valence levels using k-nearest neighbors (KNN) (k = 3). The features used with the classifier were extracted from the DEAP dataset by dividing EEG samples into several overlapping windows with widths between 2 s and 4 s, each of which was subsequently decomposed into five frequency bands using discrete wavelet transforms. Finally, entropy and energy were computed from each of these frequency bands and served as a feature vectors.

In [35], the researchers proposed an EEG-based emotion recognition method based on empirical mode decomposition (EMD), which is a data-driven signal processing analysis technique. Their method was used to decompose each EEG signal acquired from the DEAP dataset into eight or nine oscillations on various frequency scales called intrinsic mode functions (IMFs). The first four IMFs of each signal were selected to construct feature vectors according to their contribution to emotion detection determined, using a cumulative variance contribution rate test. Feature vectors were constructed by computing the sample of each one of the IMFs for each EEG signal and then used along with SVM to perform four binary class classifications, namely HAHV/LAHV, LAHV/LALV, LALV/HALV, and HALV/HAHV. This model obtained an average classification accuracy equal to 94.98%.

In [36], the researchers used a deep neural network (DNN) to identify human emotions using the DEAP dataset. Power spectral density (PSD) and frontal asymmetry features were extracted and then fed into the DNN to classify two classes by emotion dimension (arousal and valence), achieving accuracies of 82.0%. and 48.5%, respectively. Moreover, the DNN results were compared with those of a traditional EEG signal classification method, namely random forest (RF) classification, achieving a 48.5% classification accuracy.

In [37], PSD features were extracted in both time and frequency domains. Then, authors proposed the application of a deep convolutional neural network (deep CNN model) in emotion recognition and obtained accuracy of 88.76% and 85.75% on valence and arousal dimension, respectively.

Table 1 presents a comparison of the abovementioned studies with respect to the EEG dataset they acquired, the number and types of extracted features they computed, the number and types of classifiers they used, and the optimal accuracies they obtained.

A considerable number of researchers have taken a further step beyond conventional machine learning techniques to find promising concepts and methods for enhancing the performance of EEG-based emotion detection systems.

In [38], the researchers built an EEG ontology using a specific set of features, and then performed feature reduction by choosing the features most relevant to the emotional state using Spearman correlation coefficients. These coefficients were calculated between the EEG features and two emotional dimensions (arousal and valence). An analysis of variance (ANOVA) test was used to search for differences in activation on different arousal–valence levels. These two tests were computed on the EEG ontology, and then the most relevant features were selected to finalize the classification process with the C4.5 algorithm.

In [39], the authors proposed a special structure for an emotion detection system that builds and trains a separate classifier (SVM) for each EEG channel feature vector, as opposed to building one classifier and training it through the combination of all the channels’ feature vectors. A weighted fusion of all these classifiers was used to detect the desired emotional label. This structure was suggested based on several studies that proved that the intensity at which emotional information is processed differs according to different brain areas.

Because of SNNs’ fast processing of information [5], they have been used in studies as a classifier, trained using an EEG dataset for various purposes. For example, in [40], an accuracy of 93.28% was achieved using an SNN and delta content extracted by wavelet transform from an EEG dataset, which was collected using the P3 Speller paradigm to classify P300 signals. These results showed that the SNN outperformed other algorithms used on the same dataset. In a recent study [41], the authors proposed using a spiking neural model (SNM) to discriminate EEG signals from motor imagery movements when it is necessary to avoid a long calibration session.

The use of NeuCube, which is an SNN architecture developed for spatio- and spectro-temporal brain data and proposed in [7], encouraged the researchers in [5] to conduct classification experiments based on different EEG datasets. A NeuCube-based SNN classifier was used in [8] to investigate the attention bias of consumers when a specific marketing stimulus was presented by classifying each EEG sample in a dataset of 90 total samples into five classes: alcoholic, nonalcoholic, design, drink color, and brand name. The classifier obtained an accuracy of 90%, which is superior to that obtained by MLP, MLR, and SVM. GO/NOGO cognitive patterns were measured in [42] based on EEG and using a NeuCube-based SNN as a classifier trained on 42 EEG samples. The classifier achieved an accuracy of 90.91%, which was superior compared with other traditional classifiers.

In recent literature, unsupervised novelty detection using SNN approach is proven to have high effectiveness [31,43]. Researchers reported that the SNN approach has a high potential to be explored and used to detect and recognize human cognitive and affective states. In [43], the SNN approach has been used on EEG signals processing and classification, They developed a model to use EEG data in order to provide insight into brain function in depression and to study the neurobiological characteristics of depressed individuals who respond to mindfulness.

In another recent publication [31], researchers built a SNN model for recognizing EEG data and classifying emotional states. Their experimental results showed that the SNN achieved the highest accuracy compared to other conventional approaches due to its processing capability for spatial and temporal data. In their experiments, they used two datasets, DEAP and SEED datasets. With the DEAP dataset, their model achieved accuracies of 74% on arousal, 78% on valence, 80% on dominance, and 86.27% on liking. With the SEED dataset, their model achieved an overall accuracy is 96.67%.

From the above-mentioned review, we concluded as follows. (i) The size of the datasets used in most of these studies was no smaller than 300 EEG samples. For example, in the DEAP dataset, 32 subjects participated in the EEG-recording experiments, in which each had an EEG sample recorded for each 40-s music clip stimulus. (ii) Feature extraction in most of these studies was not a minor task in terms of the number of features as well as the processes underlying their extraction, which emphasizes the need for a sufficient EEG dataset and effective feature extraction method for the accurate classification of emotions from EEG. (iii) EEG-based emotion detection is one of the EEG-based pattern recognition problems. These problems have been solved by several useful machine learning algorithms, and one promising algorithm that has yet to be investigated is SNN. (iv) SNN models have been developed effectively to enhance the analysis and understanding of spatio-temporal brain data and recognize cognitive and affective states. These reasons encouraged us to build an EEG-based emotion detection system with a smaller version of the DEAP dataset using a NeuCube-based SNN as a classifier.

## 4. Proposed System

**Figure 5:**
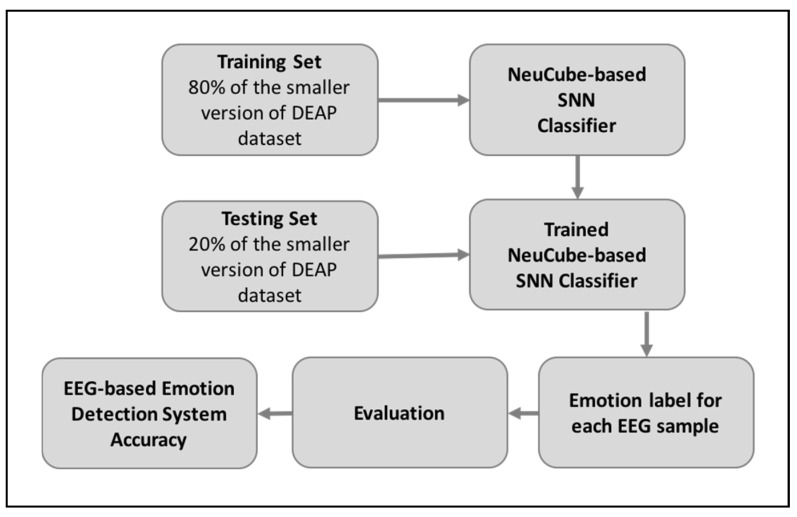
The proposed solution.

In this section, we illustrate our methodology along with implementation details of our proposed solution. It consists of three stages: the construction of smaller versions of the DEAP dataset, classification with the NeuCube-based SNN classifier, and evaluation of the trained classifier’s accuracy. Figure 5 shows the structure of the proposed solution model. Using this model, we performed four experiments, each of which used different sample sizes.

### 4.1. Dataset

The DEAP dataset is a multimodal benchmark dataset for the analysis of human affective states [44]. The EEG and peripheral physiological signals of 32 participants were recorded as each watched 40 1-min excerpts of music videos. Participants rated each video in terms of the levels of arousal, valence, like/dislike, dominance, and familiarity.

The sampling rate of the original recorded EEG data of 512 Hz was down-sampled to a sampling rate of 128 Hz with a bandpass frequency filter that ranged from 4.0–45.0 Hz, and the electrooculogram (EOG) artifacts were eliminated from the signals using a blind source separation method, namely independent component analysis (ICA).

In this study, we injected the preprocessed data directly into the learning model without being subjected to any feature extraction or selection process. The two EEG channels of FP1 and FP2 obtained good accuracy in [34], as did F3 and C4 in [35]; thus, we only used FP1, F3, C4 and C3 channels as the raw features of the dataset. We selected the EEG data points according to the accuracy achieved in [35] by selecting 1152 EEG data points from 34 s to 42 s, and related this selection to the participants’ emotional stability in this segment of the 1-min music video stimuli.

We constructed four datasets with two different sizes, 60 and 40 samples, and we performed two binary classifications, one for valence levels and one for arousal levels, for each of these datasets to enable us to measure the classification accuracy corresponding to the dataset size. We performed four experiments, each of which was performed using one of the smaller-version datasets constructed from the DEAP dataset.

Experiment 1 (60-Exp1) used a 60-sample dataset constructed by selecting the first 10 trial samples of the first six participants, whereas Experiment 2 (40-Exp2) used a 40-sample dataset constructed by selecting the first 10 trial samples of the first four participants. Experiment 3 (60-Exp3) used a 60-sample dataset constructed by selecting 10 trial samples, from the eleventh trial to the twentieth trial, of six participants, starting from the seventh participant to the twelfth participant, whereas Experiment 4 (40-Exp4) used a 40-sample dataset constructed by selecting the first 40 samples of the second 60-sample dataset.

The EEG-based emotion classification proposed in this study was conducted according to the valence and arousal dimensions, with each dimension divided into high (ratings of 5–9) and low (ratings of 1–5) classes; thus, two binary classifications of valence and arousal were included in each of the four experiments.

Table 2 summarizes the description of the original DEAP dataset and this study’s datasets, which were constructed from the original DEAP dataset.

### 4.2. NeuCube-Based SNN Classifier

We performed our experiments using NeuCube v1.3 software implemented in Matlab. The NeuCube is a modular development system for SNN applications on spatio- and spectro- temporal data. It was developed by the Knowledge Engineering and Discovery Research Institute (KEDRI, www.kedri.aut.ac.nz). NeuCube is based on the evolving connectionist system (ECOS) principles and neuromorphic computations. It facilitates building spatiotemporal data machines (STDM) for problem solving such as classification, prediction, pattern recognition, data analysis and data understanding.

NeuCube includes modules for data encoding, unsupervised learning, supervised classification and regression, visualization, pattern discovery, and model optimization. NeuCube facilitates the building of SSN applications, using the following steps: input data transformation into spike sequences; mapping input variables into spiking neurons; deep unsupervised learning spatio-temporal spike sequences in a scalable 3D SNN reservoir; on-going learning and classification of data over time; dynamic parameter optimization; evaluating the time for predictive modelling; adaptation on new data, possibly in an on-line/real time mode; model visualization and interpretation for a better understanding of the data and the processes that generated it; implementation of a SNN model: von Neumann vs neuromorphic hardware systems.

**Figure 6:**
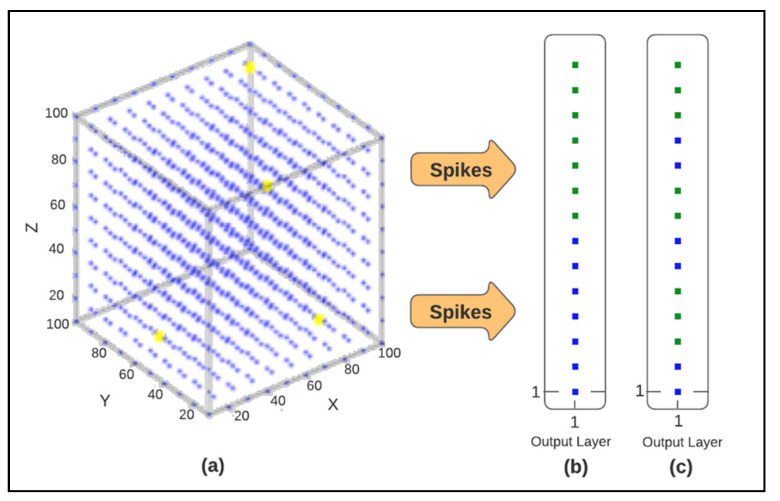
Architecture of the Spiking neural networks (SNN) cube trained using 60 samples (**a**), with the output layer colored by the true (**b**) and predicted (**c**) labels of the samples.

The NeuCube-based classifier was built using the following four steps:

1. Data were encoded into spikes using the threshold-based representation (TBR) method with a 0.5 threshold.
2. The SNN cube was initialized with 1000 spiking neurons and a 2.5 small-world radius, which defined the distance that constrained the number of each neuron’s initial connections to its neighboring neurons.
3. Unsupervised training of the cube was performed using a threshold for neuron firing = 0.5 and unsupervised STDP learning rate = 0.01.
4. The output layer classifier was classified with a modulation factor = 0.8 and drift value = 0.005. An architecture of the SNN cube trained using 60 samples is shown in Figure 6a, with the output layer when colored by true labels shown in Figure 6b, and when colored by predicted labels shown in Figure 6c.

We assigned the NeuCube parameters as the values of the original NeuCube model, but further parameter optimization could lead to better accuracy.

For all the experiments, we divided the dataset into 80% training set and 20% testing set. The 20% of the dataset represent a testing set that has not been used during the training phase and the results are based on these new unseen samples which shows that the model could be generalized to a new subject. We evaluated our system performance by the accuracy of the classification in terms of the number of correctly classified emotions based on the EEG sample over all EEG samples in the testing set.

## 5. Results and Discussion

We evaluated the proposed system accuracy using four smaller versions of the DEAP dataset by performing a binary classification according to the valence dimension and a binary classification according to the arousal dimension for each version of the dataset. The results of the four experiments are listed below:

60-Exp1: A 60-sample EEG dataset was used to train and test the proposed system, obtaining an accuracy of 66.67% for valence classification and 69.23% for arousal classification. The accuracy of the low valence class was 0%, whereas that of the high valence class was 88.89%. Furthermore, the accuracy of the low arousal class was 100%, whereas that of the high arousal class was 33.33%.

40-Exp2: A 40-sample EEG dataset was used to train and test the proposed system, obtaining an accuracy of 55.56% for valence classification and 66.67% for arousal classification. The accuracy of the low valence class was 0%, whereas that of the high valence class was 83.33%. Furthermore, the accuracy of the low arousal class was 83.33%, whereas that of the high arousal class was 33.33%.

60-Exp3: A 60-sample EEG dataset was used to train and test the proposed system, obtaining an accuracy of 84.62% for valence classification and 61.54% for arousal classification. The accuracy of the low valence class was 0%, whereas that of the high valence class was 100%. Furthermore, the accuracy of the low arousal class was 57.14%, whereas that of the high arousal class was 66.67%.

**Figure 7:**
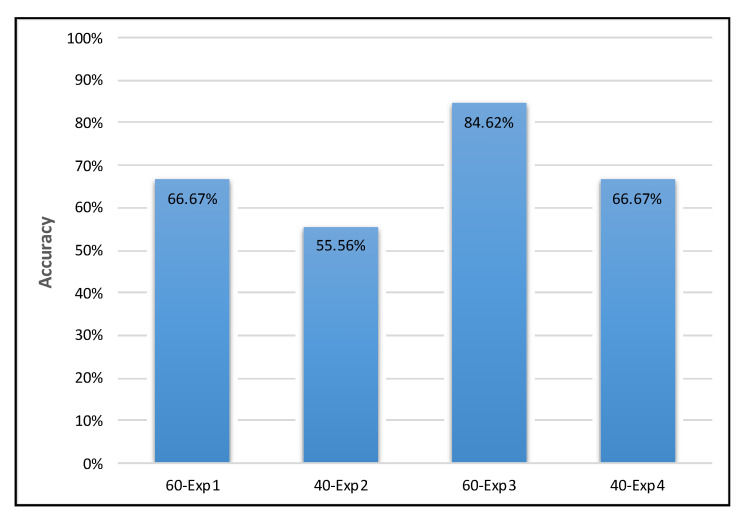
Comparison of the valence experiments’ results.

**Figure 8:**
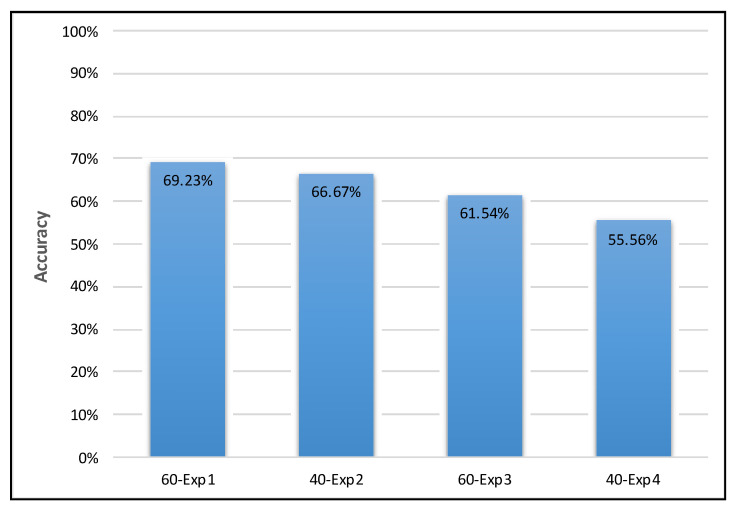
Comparison of the arousal experiments’ results.

40-Exp4: A 40-sample EEG dataset was used to train and test the proposed system, obtaining an accuracy of 66.67% for valence classification and 55.56% for arousal classification. The accuracy of the low valence class was 0%, whereas that of the high valence class was 85.71%. Furthermore, the accuracy of the low arousal class was 60%, whereas that of the high arousal class was 50%. Figure 7 presents a comparison of the valence experiments’ results; Figure 8 presents a comparison of the arousal experiments’ results; and Table 3 presents a comparison of all the experiments in both dimensions.

The classification accuracy obtained in all experiments ranged between 56% and 85%, as shown in Figure 7 and Figure 8, which is comparable to that obtained in previous studies that used the same dataset for EEG-based emotion detection.

We used two EEG dataset sizes (60, 40), which were smaller than the datasets used in previous studies, as shown in in Table 3; however, we were able to obtain an accuracy comparable to those studies that used large versions of the same dataset.

These results demonstrate the potential ability of NeuCube-based SNNs as well as SNNs in general to learn to detect emotion from EEGs with a small EEG dataset and to learn relevant patterns in raw EEG datasets without any need to extract the features manually. Using a NeuCube-based SNN in an EEG-based emotion detection system may not improve classification accuracy significantly, but it provides an option for a building process for an EEG-based emotion detection system that is lightweight in terms of a small EEG sample size and with no feature extraction processes involved.

**Figure 9:**
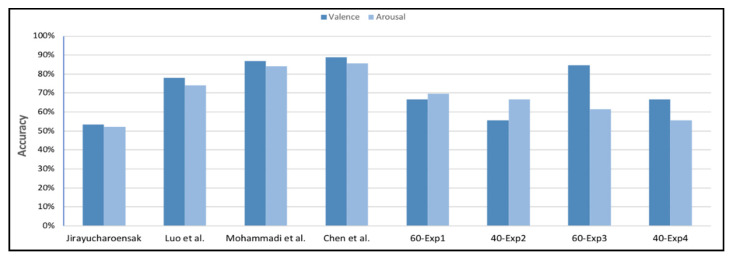
Comparison with other studies that used the DEAP dataset.

In previous studies, the extraction of relevant features from the EEG dataset involved several complex methods. By contrast, in this study, we did not include any feature extraction method and fed the classifier directly with the EEG dataset; however, we still obtained an accuracy comparable to other studies that used several feature extraction methods on the same dataset, as shown in Table 4 and Figure 9.

We also tested some state-of-the-art traditional machine learning classifiers which included Naïve Bayes, Bayesian Network, Logistics Regression, Decision Tree, Support Vector Machine (SVM)and k-nearest neighbors (KNN). The average classification accuracy obtained in all experiments showed that SVM obtained the highest average accuracy of 62.71% followed by Bayesian network and KNN which achieved average accuracy of 61.88% and 61.46%, respectively.

## 6. Conclusions

BCI research studies were initially focused on applications for persons with some degree of impaired motor disability. Over the years, many EEG-based BCI emotion detection systems have been developed that targeted different application areas. They all shared the same research approach of using objective methods to determine affective emotional states. In recent times, alternative applications in healthy human study participants have increased. This later research in able-bodied humans has gained popularity for EEG-based BCI investigative research purposes.

In this study, we proposed a lightweight building process for an EEG-based emotion detection system, in terms of small EEG sample size and no feature extraction methods, using a NeuCube-based SNN. We constructed four small versions of the DEAP dataset with 40 and 60 EEG samples to perform two binary classifications according to the valence and arousal emotional dimensions. The results showed that the proposed system obtained a comparable accuracy with that of other studies that used the larger version of the same dataset with the involvement of feature extraction processes.

Although good experimental results have been achieved with our proposed NeuCube-based SNN model, further research and investigation is still needed on how to select, construct and optimize learning models to obtain a higher classification accuracy and robustness model for EEG-based emotion recognition. Therefore, we encourage the investigation of NeuCube-based SNN classifiers and other architectures of SNNs in an EEG-based emotion detection system with respect to: (i) different EEG sample sizes, (ii) different parameters, (iii) with and without a feature extraction method, and (iv) performance of multiclass classification and regression according to the different emotional dimensions.

## Figures and Tables

**Table 1:** Comparison of different conventional studies on electroencephalogram (EEG)-based emotion detection.

Ref.	Dataset	Feature Extraction	Classifier	Accuracy
[33]	Own dataset	4	Multilayer perceptron (MLP) Support Vector Machine (SVM)	82.29%.
[10]	DEAP dataset	2	Deep learning network (DLN)-100 DLN-50 DLN-50 + PCA DLN-50 + PCA + covariate shift adaptation (CSA)	53.42% (valence) 52.05% (arousal)
[34]	DEAP dataset	2	k-nearest neighbors (KNN)	86.75% (valence) 84.05 %
[35]	DEAP dataset	1	SVM	94.98%
[36]	DEAP dataset	2	Deep neural network (DNN), Random forest (RF)	82.0%, 48.5%
[37]	DEAP dataset	1	Deep convolutional neural network (CNN)	88.76% (valence) 85.75% (arousal)

**Table 2:** Description of the DEAP dataset and our smaller versions of DEAP.

Dataset /Dimension	Participants	Samples for Each Participant (Trial × Channel × Data)	Labels
Original DEAP	32	40 × 32 × 8064	1280 × 4
60-samples-Exp1	6	40 × 32 × 8064	60 × 2
40-samples-Exp2	4	40 × 32 × 8064	40 × 2
60-samples-Exp3	6	40 × 32 × 8064	60 × 2
40-samples-Exp4	4	40 × 32 × 8064	40 × 2

**Table 3:** Comparison of all experiments’ results.

Sample Size/Emotion Dimension	Valence	Arousal
60-samples-Exp1	66.67%	69.23%
40-samples-Exp2	55.56%	66.67%
60-samples-Exp3	84.62%	61.54%
40-samples-Exp4	66.67%	55.56%

**Table 4:** Comparison with other studies that used the DEAP dataset.

	Study	Dataset Subjects (Trial × Channel × Data)	Feature Extraction Methods	Accuracy	
				Valence	Arousal
	Jirayucharoensak et al. [10]	32 (40 × 32 × 8064)	2	53.42%	52.05%
	Luo et al. [31]	32 (40 × 32 × 8064)	1	78%	74%
	Mohammadi et al. [34]	32 (40 × 10 × 8064)	2	86.75 %	84.05 %
	Chen et al. [37]	32 (40 × 32 × 128)	5	88.76%	85.75%
Proposed method	60-Exp1	6 (10 × 4 × 1152)	0	66.67%	69.67%
	40-Exp2	4 (10 × 4 × 1152)	0	55.56%	66.67%
	60-Exp3	6 (10 × 4 × 1152)	0	84.62%	61.54%
	40-Exp4	4 (10 × 4 × 1152)	0	66.67%	55.56%
